# Genomic epidemiology and evolutionary dynamics of respiratory syncytial virus group B in Kilifi, Kenya, 2015–17

**DOI:** 10.1093/ve/veaa050

**Published:** 2020-07-15

**Authors:** Everlyn Kamau, James R Otieno, Nickson Murunga, John W Oketch, Joyce M Ngoi, Zaydah R de Laurent, Anthony Mwema, Joyce U Nyiro, Charles N Agoti, D James Nokes

**Affiliations:** Epidemiology and Demography Department, KEMRI–Wellcome Trust Research Programme, Kilifi, Kenya; Epidemiology and Demography Department, KEMRI–Wellcome Trust Research Programme, Kilifi, Kenya; Epidemiology and Demography Department, KEMRI–Wellcome Trust Research Programme, Kilifi, Kenya; Epidemiology and Demography Department, KEMRI–Wellcome Trust Research Programme, Kilifi, Kenya; Epidemiology and Demography Department, KEMRI–Wellcome Trust Research Programme, Kilifi, Kenya; Epidemiology and Demography Department, KEMRI–Wellcome Trust Research Programme, Kilifi, Kenya; Epidemiology and Demography Department, KEMRI–Wellcome Trust Research Programme, Kilifi, Kenya; Epidemiology and Demography Department, KEMRI–Wellcome Trust Research Programme, Kilifi, Kenya; Epidemiology and Demography Department, KEMRI–Wellcome Trust Research Programme, Kilifi, Kenya; School of Health and Human Sciences, Pwani University, Kilifi, Kenya; Epidemiology and Demography Department, KEMRI–Wellcome Trust Research Programme, Kilifi, Kenya; School of Life Sciences and Zeeman Institute (SBIDER), University of Warwick, Coventry, UK

**Keywords:** genomes, RSV, evolution, emergence, community

## Abstract

Respiratory syncytial virus (RSV) circulates worldwide, occurring seasonally in communities, and is a leading cause of acute respiratory illness in young children. There is paucity of genomic data from purposively sampled populations by which to investigate evolutionary dynamics and transmission patterns of RSV. Here we present an analysis of 295 RSV group B (RSVB) genomes from Kilifi, coastal Kenya, sampled from individuals seeking outpatient care in nine health facilities across a defined geographical area (∼890 km^2^), over two RSV epidemics between 2015 and 2017. RSVB diversity was characterized by multiple virus introductions into the area and co-circulation of distinct genetic clusters, which transmitted and diversified locally with varying frequency. Increase in relative genetic diversity paralleled seasonal virus incidence. Importantly, we identified a cluster of viruses that emerged in the 2016/17 epidemic, carrying distinct amino-acid signatures including a novel nonsynonymous change (K68Q) in antigenic site ∅ in the Fusion protein. RSVB diversity was additionally marked by signature nonsynonymous substitutions that were unique to particular genomic clusters, some under diversifying selection. Our findings provide insights into recent evolutionary and epidemiological behaviors of RSVB, and highlight possible emergence of a novel antigenic variant, which has implications on current prophylactic strategies in development.

## 1. Introduction

Respiratory syncytial virus (RSV) is the most common cause of acute lower respiratory tract infection in children aged <5 years worldwide, with an estimated associated mortality of up to 199,000 deaths per year mostly in developing countries (Scheltema et al. 2017; Pneumonia Etiology Research for Child Health Study Group 2019). RSV is also an important cause of community-acquired pneumonia among hospitalized adults of all ages (Dowell et al. 1996). RSV has an enveloped, nonsegmented, single-stranded, negative sense RNA genome of approximately 15,000 nucleotides encoding eleven proteins: NS1, NS2, N, P, M, SH, G, F, M2-1, M2-2, and L (Sullender 2000). RSV clinical isolates are classified into two groups (RSVA and RSVB) based on antigenic and genetic variability (Melero et al. 1997). Distinct genotypes of RSV circulate locally and globally suggestive of rapid global transmission (Bose et al. 2015). The available therapeutic modalities are chiefly supportive, and prophylactic treatment with neutralizing antibodies is effective in reducing morbidity in infants (Neuzil 2016). There is no licensed vaccine for routine use in immunization, however, vaccine candidates and monoclonal antibodies (mAbs) are in advanced clinical trials (Gerretsen and Sande 2017).

We have previously characterized RSV dynamics in coastal Kenya, using the G glycoprotein gene and using whole-genome sequences of RSVA genotype ON1, almost exclusively from samples from pneumonia patients admitted to the Kilifi County Hospital (Agoti et al. 2015a; Otieno et al. 2016, 2018). From these studies, RSV displays high genetic diversity of locally circulating strains, within and between consecutive epidemics. Furthermore, recurrent RSV epidemics in Kilifi are depicted by sequential replacement of genotypes, over the long term, and high turnover of variants within genotypes in the short term (Agoti et al. 2015a; Otieno et al. 2016). In the current study, samples arise from a design aimed to limit temporal, age-related, illness severity, geographical, and health care access bias. Recruitment was carried throughout a study location, from representative health facilities, simultaneously, and of patients of any age with mild acute respiratory symptoms (Nyiro et al. 2018).

Phylodynamic methods have been used to study molecular epidemiology and evolutionary dynamics of RNA viruses including Ebola, Zika, influenza, and coronaviruses (Faria et al. 2013, 2017; Lemey et al. 2014; Dudas et al. 2017; Zehender et al. 2017; Sironi et al. 2020). However, despite the importance of RSV to pneumonia hospitalization and mortality among children (Pneumonia Etiology Research for Child Health Study Group 2019), there are few equivalent genome-scale studies to examine RSV transmission and evolution particularly within a community setting (Agoti et al. 2015b, 2017, 2019; Otieno et al. 2018; Trovao et al. 2019). While most studies on RSV focus on the G glycoprotein gene because of its high genetic diversity and utility as a phylogenetic marker, genome-wide genetic signatures additionally inform on diversity and the adaptive mechanisms following introduction into the population (Otieno et al. 2018).

We measured genomic diversity, spatial and temporal circulation of RSVB in rural Kilifi, coastal Kenya, from samples collected through outpatient surveillance, analogous to studying community RSV epidemics. We present estimates of rate of evolution and time since the most recent common ancestor (tMRCA) and infer viral population dynamics over two consecutive RSV epidemics in coastal Kenya. In particular, we identify emergence of a novel RSVB variant carrying distinct amino acid (aa) signatures.

## 2. Materials and Methods

### 2.1 Study design and sample testing

**Figure 1.**
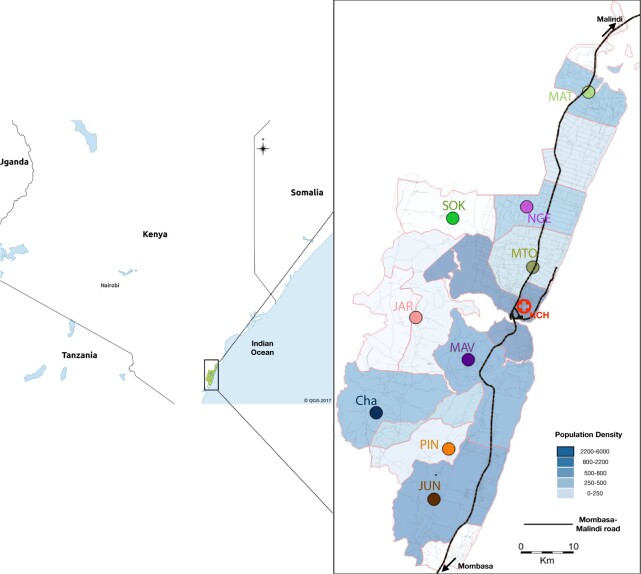
A map showing the geographical area covered in the KHDSS, expanded from a map of Kenya. The nine participating public health facilities are indicated in the map. The dark lines within the polygons indicate the road structure within KHDSS. The maps were rendered using QGIS 2.18.17 (https://www.qgis.org/).

RSV is highly seasonal in Kilifi, Kenya, starting from November through May, with a peak around January and an average duration of 18 weeks (Nokes et al. 2009). This study was carried out within the Kilifi Health and Demographic Surveillance System (KHDSS) area (Scott et al. 2012) and used samples collected from December 2015 to July 2017, a period covering two RSV seasonal epidemics (2015/16 and 2016/17). Nine public outpatient health facilities in KHDSS were purposively selected (Matsangoni (MAT), Ngerenya (NGE), Mtondia (MTO), Sokoke (SOK), Mavueni (MAV), Jaribuni (JAR), Chasimba (CHA), Pingilikani (PIN), and Junju (JUN)) to provide a broad representation covering major road networks and variation in population density (Fig. 1) (Nyiro et al. 2018). Participant recruitment and specimen collection was integrated within the routine patient care as detailed in Nyiro et al. (2018). Written individual informed consent was sought from adult patients and parents/guardians of patients below 18 years. Nasopharyngeal swabs (NPS) were screened for RSVA and RSVB using a multiplex real-time PCR assay system (Hammitt et al. 2011; Kamau et al. 2017). RSV positives were defined as samples with a cycle threshold (*Ct*) <35.0.

The study was approved by the Kenyan KEMRI-Scientific and Ethical Review Unit (SERU# 3103) and the UK University of Warwick Biomedical and Scientific Research Ethics Committee (BSREC# REGO-2015-6102).

### 2.2 RSVB whole genome sequencing and data assembly

Whole genome amplification and sequencing was attempted for all RSVB positive samples. Reverse transcription and PCR amplification were performed with a six-amplicon, six-reaction strategy presented in detail in Agoti et al. (2015b). Methods for quality checking of the sequence reads, depletion of human reads, consensus genome assemblies and calculation of coverage depth, were as described in Otieno et al. (2018).

### 2.3 Data compilation and sequence alignment

A global dataset was prepared by retrieving RSVB complete genomes from GenBank sampled between 2012 and 2016. Sequences published without date or location of sampling were excluded. For all analyses, sequence alignment was done using MAFFT v.7.221 (Katoh and Standley 2013) and the parameters ‘–localpair –maxiterate 1,000’. Because of sparse data at the genome termini and in the noncoding regions, only the coding genomic regions were used for all analyses.

### 2.4 Tests of temporal signal

A maximum-likelihood tree of the Kilifi dataset was estimated using IQ-TREE 1.6 (Nguyen et al. 2015). The best-fit substitution model was tested and chosen during the tree building process and the approximate likelihood ratio test was applied to assess the reliability of key branches of the trees (1,000 replicates). To examine the degree of temporal signal or signal of divergence accumulation over the sampling time interval, we first followed a standard exploratory linear regression approach. Root-to-tip distances were plotted as a function of sampling time according to a rooting that maximizes the Pearson product-moment correlation coefficient using TempEst (Rambaut et al. 2016).

We also explored an alternative test of temporal signal using a Mantel test (Murray et al. 2016) to identify whether genetically similar taxa were more likely to have been sampled around the same time. With this method, the significance of the correlation between the root-to-tip distances and sampling times was estimated against a null distribution obtained by reassigning the sampling dates to the taxa 1,000 times. We repeated the Mantel test incorporating a clustered permutation approach (Duchêne et al. 2015b) where dates were randomly reassigned (1,000 data replicates) among defined clusters of taxa. To identify clusters for clustered permutation, we used the patristic distances generated by IQ-TREE above and plotted a distribution plot of the distances in R (R Core Team 2014) (Supplementary Fig. S1). A cutoff value (0.0024 nucleotide substitutions per site) determined as the least value between the first and second peaks in the distribution (Supplementary Fig. S1) was used to define clusters as sequences within genetic distance threshold of <0.0024. The Mantel tests were implemented in R using Murray et al.’s scripts (Murray et al. 2016).

The significance of linear regression of sampling dates against root-to-tip distances in the clustered permutation analyses was assessed by a *P*-value: comparison of the observed data correlation coefficient (*r*) to the *r* estimates for the data replicates with dates randomly permuted over the defined clusters.

Further, we complimented the linear regression permutation tests with Bayesian dating permutations done in 100 million steps and sampling every 10,000. This involved creating ten datasets with randomly permuted tip dates as suggested in Murray et al. (2016). The significance of the Bayesian tip-date-informed evolutionary rate was determined by comparing the mean substitution rate estimate from the observed data with the 95 per cent HPDs estimated from the date randomized datasets for which no particular relationship between sampling time and root-to-tip divergence is expected while assuming the same evolutionary models in all the Bayesian dating analyses (Firth et al. 2010).

### 2.5 Bayesian analyses

Time-calibrated phylogenies were done using BEAST v1.10 (Suchard et al. 2018). To model the nucleotide substitution process, the codon positions were partitioned into 1st + 2nd vs. 3rd positions. The HKY substitution model with a discretized gamma distribution was used to model rate heterogeneity across sites (Shapiro, Rambaut, and Drummond 2006). An uncorrelated lognormal relaxed molecular clock was used to accommodate variation in evolutionary rate among lineages (Drummond et al. 2006) and a nonparametric skyride demographic model with time-aware smoothing was selected. The molecular clock rate was set to use a noninformative continuous time Markov chain rate reference prior. The analyses were done in 200 million steps, sampling every 10,000. Stationarity and mixing (e.g. based on effective sample sizes >200 for the continuous parameters) were examined using Tracer version 1.7. The Bayesian dating permutation tests (described above) were done in 100 million steps, sampling every 10,000. Maximum clade credibility (MCC) trees were generated from the BEAST posterior tree sets using TreeAnnotator.

### 2.6 Phylogeny-trait association analysis

For the Kilifi dataset alone, we used the Bayesian Tip-association Significance (BaTS) software (Parker, Rambaut, and Pybus 2008) to assess the strength of geographic clustering in the posterior tree distribution obtained from BEAST analyses. The overall statistical significance was determined by estimating the parsimony score (PS) and association index (AI) metrics, where the null hypothesis is that clustering by geographic location is not more than that expected by chance. In addition, the maximum clade (MC) size metric was used to compare the strength of clustering at each location by calculating the expected (null) and the observed mean clade size from each study location. A significance level of 0.05 was used in all cases. The PS, AI, and MC statistics were computed for a null distribution with 1,000 replicates.

### 2.7 Selection analyses

Gene-specific nonsynonymous to synonymous substitutions (dN/dS) ratios were estimated using the SLAC method (Weaver et al. 2018). We also investigated episodic positive or diversifying selection using MEME and FUBAR methods. MEME aims to detect sites evolving under positive selection in a proportion of branches, while FUBAR uses a Bayesian approach and assumes that selection pressure is constant along the entire phylogeny.

### 2.8 Sequence data availability

The sequencing reads are available in the NCBI BioProject database under the study accession PRJNA562116 and the genomes generated in this study are available in GenBank under accession numbers MN365302 to MN365600.

## 3. Results

### 3.1 RSVB occurrence in Kilifi, 2015–17

**Figure 2.**
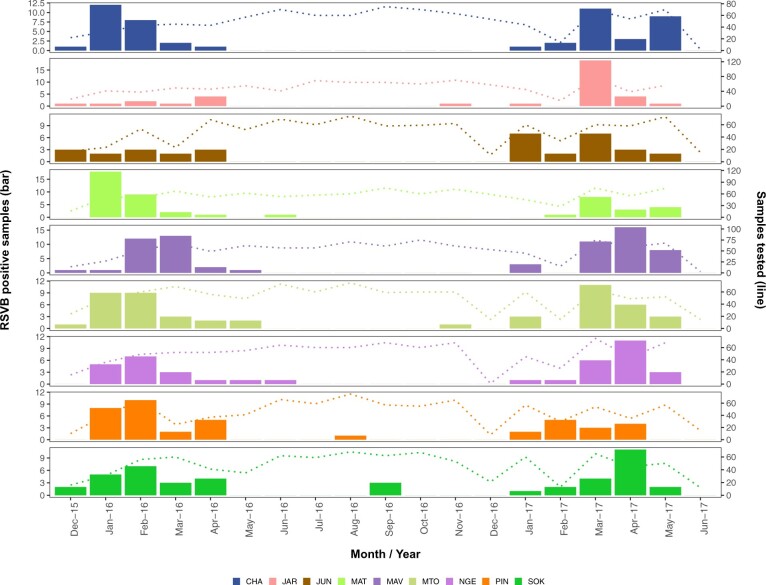
Monthly RSVB occurrence by study location: temporal and spatial distribution of RSVB positive cases (left Y axis) and number of clinical samples tested (right Y axis) from each participating health facilities. Abbreviations: CHA, Chasimba; JAR, Jaribuni; JUN, Junju; MAV, Mavueni; MAT, Matsangoni; PIN, Pingilikani; NGE, Ngerenya; SOK, Sokoke; MTO, Mtondia.

Between December 2015 and July 2017, 8,127 NPS samples were tested for RSV, and 503 (6.2%) were positive (*C_t_* < 35). Among the RSV positive samples, 95 (18.9%) were RSVA and 408 (81.1%) were RSVB. The frequency and monthly pattern of occurrence of RSVB for each participating health facility are shown in Fig. 2. Overall, the proportion of RSV positive individuals differed by age (P value <0.001) and study location (P value = 0.003) (Supplementary Table S1). The median age of RSV positive individuals was 20 months (interquartile range (IQR), 8–43 months), 81.7% (411/503) were aged 5 years or younger, and 272 (54.1%) of the cases were female (Supplementary Table S1). The peak period for RSV case detections occurred from November to May the following year.

### 3.2 Genome characteristics and relative genetic diversity

**Figure 3.**
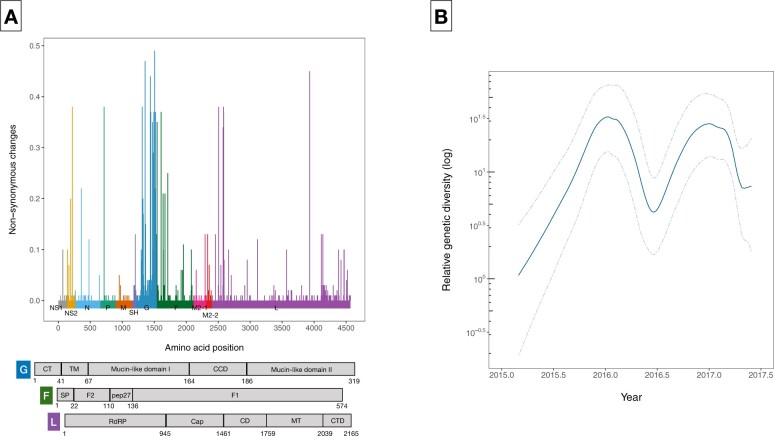
(A) Relative frequencies of potential nonsynonymous changes across codon-aligned RSV genome sequences. The frequencies for each codon position are calculated as the number of nonsynonymous nucleotide substitutions for all pairwise comparisons in a sequence alignment, while excluding ambiguous bases or insertions. Abbreviations: CT, cytoplasmic; TM, transmembrane; CCD, central conserved domain; SP, signal peptide; RdRp, RNA-dependent RNA polymerase; Cap, capping; and MT, methyltransferase; CD, connector domain; CTD, C-terminal domain. (B) Bayesian skygrid analysis depicting fluctuating relative genetic diversity for the two RSV epidemics. Solid line represents mean relative genetic diversity while the corresponding dashed lines indicate the 95 per cent HPD intervals.

Sequencing and data assembly was successful for 299/408 (73.3%) RSVB positive samples. The remaining 26 per cent (109/408) were not sequenced at sufficient depth or the read quality was low. The final dataset of the Kilifi RSVB samples consisted of 295 coding-complete genomes. The median genome length was 15,025 (range 11,519–15,257 nt). All the sequenced RSVB viruses belonged to the BA genotype, characterized by the presence of 60-nt duplication in the C-terminal region of the G glycoprotein gene. Genome coverage did not vary by rRT-PCR *C_t_* value. Across the genome length, there were 838 consensus level single nucleotide polymorphisms: 554/838 (66%) were parsimony informative, 503/554 (91%) were located within coding regions, and 332/503 (66%) were nonsynonymous. Nonsynonymous changes were higher at the mucin-like domains of G gene; in the N-terminal of fusion (F) gene; as well as in the N- and C-terminals of RNA-dependent RNA polymerase (L) gene (Fig. 3A).

Bayesian demographic reconstruction (Fig. 3B) showed seasonal periodicity in relative genetic diversity corresponding with RSVB incidence. Such temporal resolution of changes in the viral population size could imply sufficient sampling density (Otieno et al. 2016). A decline in relative genetic diversity was observed between the two epidemics indicating lineage or variant replacement.

### 3.3 Phylogenetic relationships and spatial structure

**Figure 4.**
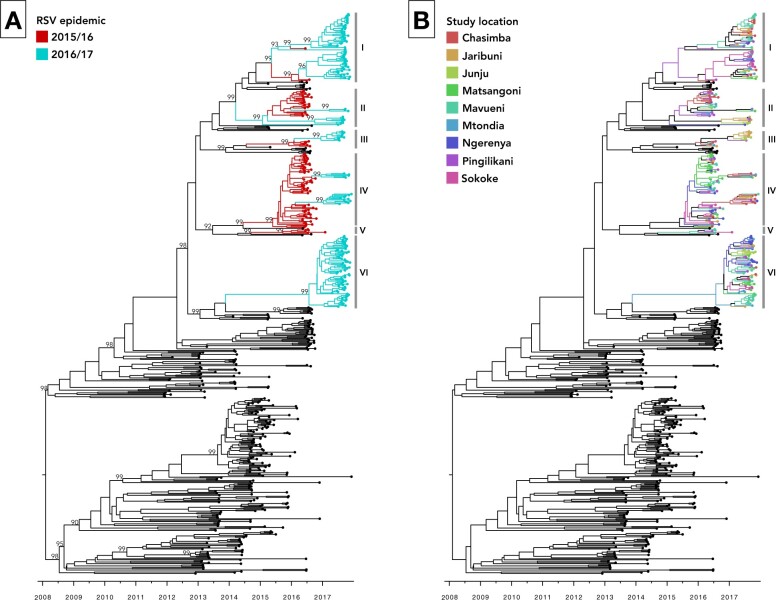
MCC phylogenies inferred for 573 viruses sampled globally between 2012 and 2017. (A) Temporal structure of the Kilifi genomes with tip colors indicating the sampling period (RSV epidemic). Clade assignments are indicated as I to VI, similarly to Supplementary Table S2. Node support is indicated by posterior probability values. **(**B**)** MCC tree similar to (A) but showing the spatial patterns of the RSVB introductions in Kilifi, Kenya with tips indicating the sampling location. In both phylogenies, sequences from outside Kilifi are colored black.

We identified six well supported clades based on the phylogenetic positioning of the Kilifi samples in the context of globally sampled RSVB genomes (Fig. 4A). Three clades (II, III, and IV) further segregated into defined temporal sub-clades. The absence of external sequences nested within the Kilifi clades might suggest local persistence and diversification, although we cannot exclude importation events from unsampled locations. Distinctively, clades V and VI solely contained viruses from the 2015/16 and 2016/17 epidemics, respectively, while the other clades contained samples from both RSV epidemics. For each clade, we estimated the duration or persistence based on sample collection dates and the time of divergence (Supplementary Table S2), but these inferences might be biased due to assorted sampling locally and globally.

The inferred clades were spatially disseminated (Fig. 4B), suggesting frequent mixing within Kilifi, and none was restricted to a single study location. We calculated AI and PS values statistics to assess the strength of spatial association with the RSVB transmission patterns in Kilifi. The analysis showed high AI and PS values (Table 1), suggesting strong and significant spatial structuring for each location (*P *<* *0.001). Geographic clustering was significant in at least 8/9 study locations as shown by the MC size values (Table 1). Differences in the observed and expected MC values (Table 1) suggested that Mavueni exhibited the most spatial structure (difference of 8.7) and Mtondia had the least (difference of 0.3).

**Table 1.** Results of Bayesian analysis of phylogeographic structure of RSVB viruses in Kilifi, coastal Kenya, 2015–17.

	Association index (AI)			Parsimony score (PS)			Maximum clade size			
	**(95% CI)** ^a^			**(95% CI)** ^a^			**(95% CI)** ^b^			
Location	Observed	Expected	*P*-value	Observed	Expected	*P*-value	Observed	Expected	*P*-value	**Difference** ^c^
ALL	14.8 (13.8–15.8)	31 (29.5–32.3)	<0.001	132.6 (129–136)	208.4 (202–214.2)	<0.001	–	–	–	–
CHA	–	–	–	–	–	–	4.6 (4–6)	1.84 (1.1–2.7)	10E-4	2.62
JAR	–	–	–	–	–	–	5.27 (5–6)	1.56 (1–2.08)	10E-4	3.71
JUN	–	–	–	–	–	–	6 (6–6)	1.55 (1–2.03)	10E-4	4.45
MAT	–	–	–	–	–	–	6.32 (4–10)	1.71 (1–2.2)	10E-4	4.61
MAV	–	–	–	–	–	–	11 (11–11)	2.3 (1.76–3)	10E-4	8.7
MTO	–	–	–	–	–	–	2 (2–2)	1.7 (1–2.3)	0.21	0.3
NGE	–	–	–	–	–	–	3.65 (2–4)	1.64 (1–2.2)	10E-4	2.01
PIN	–	–	–	–	–	–	3.1 (3–4)	1.73 (1–2.2)	0.0084	1.37
SOK	–	–	–	–	–	–	4 (3–5)	1.8 (1.1–2.3)	10E-4	2.2

*P* values correspond to the proportion of trees from the expected (null) distribution equal to, or more extreme than, the median posterior of the statistic. Abbreviations: CHA, Chasimba; JAR, Jaribuni; JUN, Junju; MAV, Mavueni; MAT, Matsangoni; PIN, Pingilikani; NGE, Ngerenya; SOK, Sokoke; MTO, Mtondia.

a AI and PS metrics were determined for all locations combined.

b MC size was determined for each specific location.

c Difference between observed and expected (null) clade size.

### 3.4 Temporal signal and molecular dating

**Figure 5:**
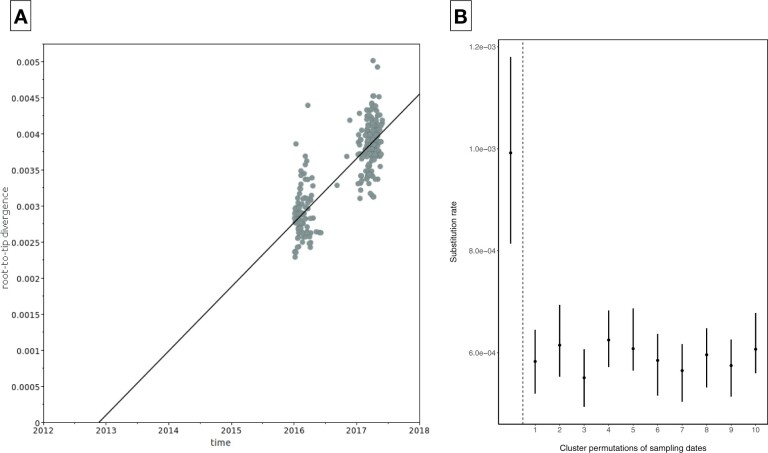
(A) Root-to-tip genetic distances as a function of sampling time. (B) Estimates of the mean and 95 per cent HPD interval of the substitution rate (substitution/site/year) from the real/actual dataset (bordered by dashed line), and from the ten datasets generated by clustered permutation of sampling dates.

The standard linear regression exploration of the Kilifi dataset showed an overall correlation between the root-to-tip distances and time (correlation coefficient of 0.85), and a clear difference in root-to-tip distances between the two RSV epidemics (Fig. 5A). A Mantel test (Murray et al. 2016) applied to the Kilifi dataset however found evidence of significant confounding between temporal and genetic structures (*P *=* *0.001) and indicated that an alternative approach to date randomization (clustered permutation) should be used to test for temporal structure. For this, we grouped the Kilifi genomes into twenty clusters using the pairwise patristic distance threshold of 0.0024 (see Section 2) and repeated the Mantel test with sampling dates permuted over the twenty clusters (1,000 permutations). The Mantel test after clustering confirmed that our choice of clusters was sufficient to eliminate the confounding (*P *=* *0.98).

To assess the significance of the correlation between phylogenetic root-to-tip distance against sampling time, we performed a linear regression with a clustered date permutation test (100 permutations). The clustered permutation regression test reached significance (*P *=* *0.001), and the *r* estimate with the observed data was outside the range of *r* estimates obtained using date randomization (Supplementary Fig. S2), indicating that the observed correlation between the root-to-tip distances and time differs significantly from what is expected in the absence of a temporal structure (Laenen et al. 2019).

We further evaluated the presence of temporal signal with Bayesian dating permutation implemented in BEAST. Sampling dates were permuted ten times over the clusters defined earlier and the substitution rate estimates from the date-randomized datasets compared to the estimates from the true ordering of dates. The substitution rate of the true observed data was estimated as 9.922 × 10^−4^ (95% HPD: 8.14 × 10^−4–^1.18 × 10^−3^) nucleotide substitutions/site/year and did not overlap with HPD intervals obtained from the date-randomized datasets (Fig. 5B). This indicates an appreciable presence of temporal signal and structure in the data. The tMRCA for the Kilifi samples was estimated to 2012 (95% HPD: 2011.1–2013.5).

### 3.5 Amino acid diversity

An alignment of aa sequences showed mutations characteristic to each clade (Supplementary Table S2). In the two RSV epidemics, there were at least 37 and 93 aa substitutions in F and G gene, respectively. In F gene, three aa substitutions (A103V, L172Q, and S173L) were found in antigenic site V in the 2015/16 viruses. These three substitutions were also circulating from 2015 to 2017 in the USA (Bin et al. 2019) and South Africa (Liu et al. 2020), and in both countries, the polymorphisms were detected continuously and in increasing frequency. However, none of the samples from the 2016/17 epidemic in Kilifi had A103V, L172Q, and S173L substitutions probably due to removal by purifying selection. It was suggested that the three substitutions likely emerged in 2014/15 as they were not present in RSV F sequences prior to 2013 (Bin et al. 2019).

Numerous nonsynonymous substitutions in G gene occurred together discriminating the two epidemics (Supplementary Fig. S3): including R136T, N144H, R260G, T279I, P289L, and K312R that characterized viruses circulating in the 2015/16 epidemic; and Y90H, L91F, P101S, T225N, T273I, and H285Y that characterized viruses in 2016/17. Additional clade-specific aa variations identified in other genomic regions are listed in Supplementary Table S2.

Six distinct aa variants—K68Q in F gene; Y90H, L91F, T225N, T273I, and A301T in G gene—occurred together at a frequency >40 per cent in the 2016/17 epidemic and distinctively characterized the sixth clade (Fig. 4A). Importantly, the K68Q substitution is found at the antigenic site ∅ of pre-fusion F protein conformation, a binding epitope of mAb MEDI8897 (Zhu et al. 2017). A variant with the mutation K68N was reported in 2 per cent of sampled viruses circulating in 2015–16 in the USA (Bin et al. 2019). In addition, we found that 32 per cent of the RSVB positive samples collected from inpatient admissions (<59 months) at the Kilifi County Hospital (a referral facility serving a larger catchment area in Kilifi county), in the 2016/17 epidemic, clustered with the G gene sequences from the sixth clade (data not shown). These inpatient RSVB strains similarly contained the aa substitutions Y90H, L91F, T225N, T273I, and A301T in G gene, suggesting wider circulation of this variant.

### 3.6 Selection pressure analyses

We estimated higher global nonsynonymous (dN)/synonymous (dS) substitution rate ratios for G and SH glycoproteins than other genes (Table 2). SLAC analyses identified three amino-acid sites (135, 217, and 285) in G gene under significant positive selection (*P *<* *0.1). MEME analyses detected three diversifying codons in the F gene, and eleven in the G gene (*P *<* *0.1) (Table 2). The FUBAR method identified two codon sites in F gene and seven in G gene, under episodic positive selection with significant support (posterior probability >0.9) (Table 2).

**Table 2.** The predicted nature of selection pressures acting on each genomic region: first column shows the computed mean dN/dS rate ratio using SLAC and the second column shows amino-acid sites in F and G gene under episodic selection as identified by MEME analyses.

Nonsynonymous (dN)/synonymous (dS) substitution rate ratio per site		Sites subject to episodic positive/ diversifying selection
NS1	0.12	*G gene*
NS2	0.236	135, 144, 154, 172, 208, 217, 285, 291, 294, 298, 303
N	0.0832	252^a^
M	0.0525	
P	0.0642	*F gene*
F	0.179	125, 172, 173
G	0.487	
SH	0.426	
M2-1	0.264	
M2-2	0.267	
L	0.122	

Sites also detected using the FUBAR method, in addition to MEME, are underlined.

a This site was detected by the FUBAR method only.

## 4. Discussion

This study provides insights into the genomic diversity of RSVB in Kilifi county, coastal Kenya. We obtained 295 complete genomes from representative sampling across the KHDSS area, over two consecutive RSV epidemics. The two epidemics comprised of multiple co-circulating virus clade introductions, which circulated in all the study locations, suggesting substantial spatial spread and transmission in a relatively short time. Although RSV surveillance has improved globally, complete genomes data from recent years remains insufficient and may have limited our inference of spatial and temporal placement of RSVB in Kilifi. In the two epidemics, there was a strong spatial structure of the viral population indicating local transmission within the populations neighboring a health facility.

Tip-dated inferences are reliable only if the sequence data exhibits temporal signal (Drummond et al. 2006; Firth et al. 2010). Conventionally, this is based on the fit of linear regression between sampling time and root-to-tip distance, and assuming statistical independence, a significant positive correlation would indicate presence of evolutionary change within the dataset timescale (Drummond, Pybus, and Rambaut 2003; Drummond et al. 2006). However, linear regression tests alone can be misleading when there is substantial rate variation among lineages, non-uniform distributed sampling times (Rieux and Balloux 2016), imbalanced trees and confounding temporal and genetic structures (i.e. closely related sequences arising from sampling at similar times, e.g. during an outbreak) (Duchêne, Duchêne, and Ho et al. 2015a; Duchêne et al. 2015b). Date randomization tests whether the observed tip-date informed estimates deviate from estimates obtained in absence of temporal structure (Duchêne et al. 2015b). The Kilifi dataset was temporally and genetically confounded. However, the temporal signal in the data remained when the clustered date permutation approach was used confirming detectable temporal structure and assuring reliability of the observed tip-dated substitution rate and tMRCA inferences. Confounding may have arisen naturally from our clinical sampling protocol or from the evolutionary process itself, as suggested previously (Murray et al. 2016). According to Vrancken et al. (2020), intense sampling of closely related sequences produces a rapid succession of coalescent events just before sampling, reminiscent of a panmictic population that is declining in size and in turn biases the evolutionary rate estimate and results in misleadingly recent tMRCA (Vrancken et al. 2020). An evolutionary change in the genetic constitution of a virus population could lead to sequences sampled synchronously being more closely related, for instance in the ‘ladderized’ Influenza A genealogies, hence inherent temporal and genetic confounding (Murray et al. 2016).

A previous study showed that K68N substitution in F gene affected binding of MEDI8897, an RSV pre-F-specific human mAb under clinical evaluation as a passive immunization of all infants entering their first RSV season (Domachowske et al. 2018; Zhu et al. 2018). It is probable that the K68Q substitution identified in clade VI in Kilifi promoted evasion of pre-existing immune responses. Unexpectedly, in our data, the F gene aa position 68 was not under detectable selection pressure. An explanation would be the low rate of nonsynonymous evolution (conversely, high sequence conservation) at position 68 in our dataset, or immune driven positive selection could not be identified by methods here. In any case conventional approaches for measuring selection pressure consistently detect positive selection only at codon sites with high rates of nonsynonymous evolution (Kosakovsky Pond and Frost 2005).

Our study provides a novel sequence polymorphism (K68Q) within the MEDI8897 binding site with a frequency of nearly 50 per cent in our study population. Additionally, the viruses with the K68Q mutation carried five distinctive amino-acid mutations in G gene, including two consecutive codons (Y90H and L91F). We are not certain whether these codon replacements are due to nonselective epidemiological processes or are compensatory mutations that retain protein function, or hitchhikers carried along by chance (Smith et al. 2004). Still, we cannot exclude the possibility that these are relevant antigenic epitopes.

In conclusion, we present the utility of genomic analyses to investigate virus transmission and genetic diversity including detection of a novel antigenically distinct variant. Further studies are required to determine whether the K68Q mutation is adaptive and/or a result of escape from antibody-mediated selection and constitutes a naturally acquired antiviral resistance that disrupts neutralizing antibody recognition and binding. Our study underscores the need for continued genomic surveillance of F and G protein antigenic sites as this has implications on RSV therapeutic and vaccine development. An important future effort for us is to assess if the K68Q mutation has become more prevalent and gradually fixed since the 2016/17 epidemic. Additional sequencing of RSVB from other regions in Kenya and neighboring countries is also essential to refine evolutionary dynamics and draw better conclusions about geographic origins of viral introductions in Kilifi. The present study makes publicly available a large number of newly sequenced RSVB genomes useful for further molecular evolution studies.

### Data Availability

The replication data and analysis scripts for this manuscript are available from the Harvard Dataverse: DOI: https://doi.org/10.7910/DVN/RYISUE. Some of the clinical dataset contains potentially identifying information on participants and is stored under restricted access. Requests for access to the restricted dataset should be made to the Data Governance Committee (dgc@kemri-wellcome.org).

## Supplementary Material

veaa050_Supplementary_Data
