# Supplementary material for: Genomic epidemiology and evolutionary dynamics of respiratory syncytial virus group B in Kilifi, Kenya, 2015–17
Source: Virus Evol. 2020 Jul 15;6(2):veaa050. doi: 10.1093/ve/veaa050 (PMC7474930; doi:10.1093/ve/veaa050)
Supplement: veaa050_Supplementary_Data [file ve_6_2_veaa050_s6.zip › SuppTable1.docx]

**Supplementary Table 1**

Distribution of RSV positive and negative samples by age, gender, and participating outpatient health facilities between December 2015 and June 2017

| **Characteristic** | **Virus positive samples** | **%** | **Virus negative samples** | **%** | **Total (n)** | ***P* value** |
| --- | --- | --- | --- | --- | --- | --- |
|  | **(n=503)** | 6.19 | 7624 | 93.8 | 8127 |  |
| **Age in years** |  |  |  |  |  |  |
| Mean | 9 |  | 149.4 |  | 144.0 |  |
| Median (IQR) | 20 (8-43) |  | 54(18-194) |  | 49 (17-188) |  |
| **Sex** |  |  |  |  |  |  |
| Male | 231 | 6.7 | 3195 | 93.3 | 3426 | 0.078 |
| Female | 272 | 5.8 | 4428 | 94.2 | 4700 |  |
| **Age Category** |  |  |  |  |  |  |
| 0–5 mo | 85 | 11.3 | 665 | 88.7 | 750 | <0.001 |
| 6–11 mo | 84 | 11.0 | 681 | 89.0 | 765 |  |
| 12–23 mo | 102 | 9.1 | 1021 | 90.9 | 1123 |  |
| 24–35 mo | 76 | 9.6 | 712 | 90.4 | 788 |  |
| 3–4 y | 64 | 6.7 | 898 | 93.4 | 962 |  |
| 5–9 y | 37 | 3.4 | 1046 | 96.6 | 1083 |  |
| 10–19 y | 21 | 2.0 | 1024 | 98.0 | 1045 |  |
| 20–49 y | 23 | 2.1 | 1098 | 98.0 | 1121 |  |
| 50–100 y | 11 | 2.3 | 479 | 97.8 | 490 |  |
| **Health Facility** |  |  |  |  |  |  |
| Matsangoni | 58 | 6.1 | 895 | 93.9 | 953 | 0.003 |
| Ngerenya | 58 | 6.5 | 840 | 93.5 | 898 |  |
| Sokoke | 50 | 5.7 | 826 | 94.3 | 876 |  |
| Mtondia | 66 | 6.9 | 898 | 93.2 | 964 |  |
| Mavueni | 79 | 8.6 | 838 | 91.4 | 917 |  |
| Jaribuni | 43 | 5.1 | 803 | 94.9 | 846 |  |
| Chasimba | 67 | 7.5 | 826 | 92.5 | 893 |  |
| Junju | 40 | 4.3 | 889 | 95.7 | 929 |  |
| Pingilikani | 42 | 4.9 | 809 | 95.1 | 851 |  |
