# Supplementary material for: Genomic epidemiology and evolutionary dynamics of respiratory syncytial virus group B in Kilifi, Kenya, 2015–17
Source: Virus Evol. 2020 Jul 15;6(2):veaa050. doi: 10.1093/ve/veaa050 (PMC7474930; doi:10.1093/ve/veaa050)
Supplement: veaa050_Supplementary_Data [file ve_6_2_veaa050_s6.zip › SuppTable2.docx]

**Supplementary Table 2**

Number of samples, time of divergence and duration of the identified RSVB clades in Kilifi.

Also shown are amino acid substitutions identified in majority of sequences in the respective clades. 95% credible intervals are indicated in the brackets.

| Clade | No. of samples | tMRCA | Duration of persistence | Amino acid substitutions in genomic regions |
| --- | --- | --- | --- | --- |
| I | 76 | May 2014 | 173 days | N gene (A97V)  G gene (P101S_,_ H267Y_,_ I268T_,_ T290A_,_ P304L)  L gene (S176N_,_ P1708L_,_ Y1980F);  F gene (I99T, I129T_,_ P104S, I5V);  NS2 gene (S53N_,_ Q101R); |
| II | 40 | September 2013 | 181 days | N gene (A97V);  G gene (N144H, I205T_,_ P214L_,_ V249A_,_ P293L_,_ S305P);  F gene (M25C);  L gene (R304Q_,_ T1956I) |
| III | 21 | January 2014 | 211 days | NS2 gene (I5T);  L gene (T1166I_,_ Q2066R);  G gene (A269V_,_ S270P_,_ E294D);  F gene (K419R) |
| IV | 80 | December 2013 | 154 days | P gene (T60I);  F gene (V103A, L172Q, S173L, I115T, P125L, T303I, I542L);  L gene (T105I_,_ Y141H_,_ Q183N);  G gene (L315P) |
| V | 5 | March 2014 | 36 days | F gene (I16T);  L gene (T1744A_,_ V1787G) |
| VI | 73 | May 2013 | 172 days | G gene (Y90H, L91F, T225N, T273I, A301T);  F gene (K68Q);  NS2 gene (K80R);  L gene (P184S) |
