# Supplementary figures and images for: Genomic epidemiology and evolutionary dynamics of respiratory syncytial virus group B in Kilifi, Kenya, 2015–17
Source: Virus Evol. 2020 Jul 15;6(2):veaa050. doi: 10.1093/ve/veaa050 (PMC7474930; doi:10.1093/ve/veaa050)

Frequency (density)

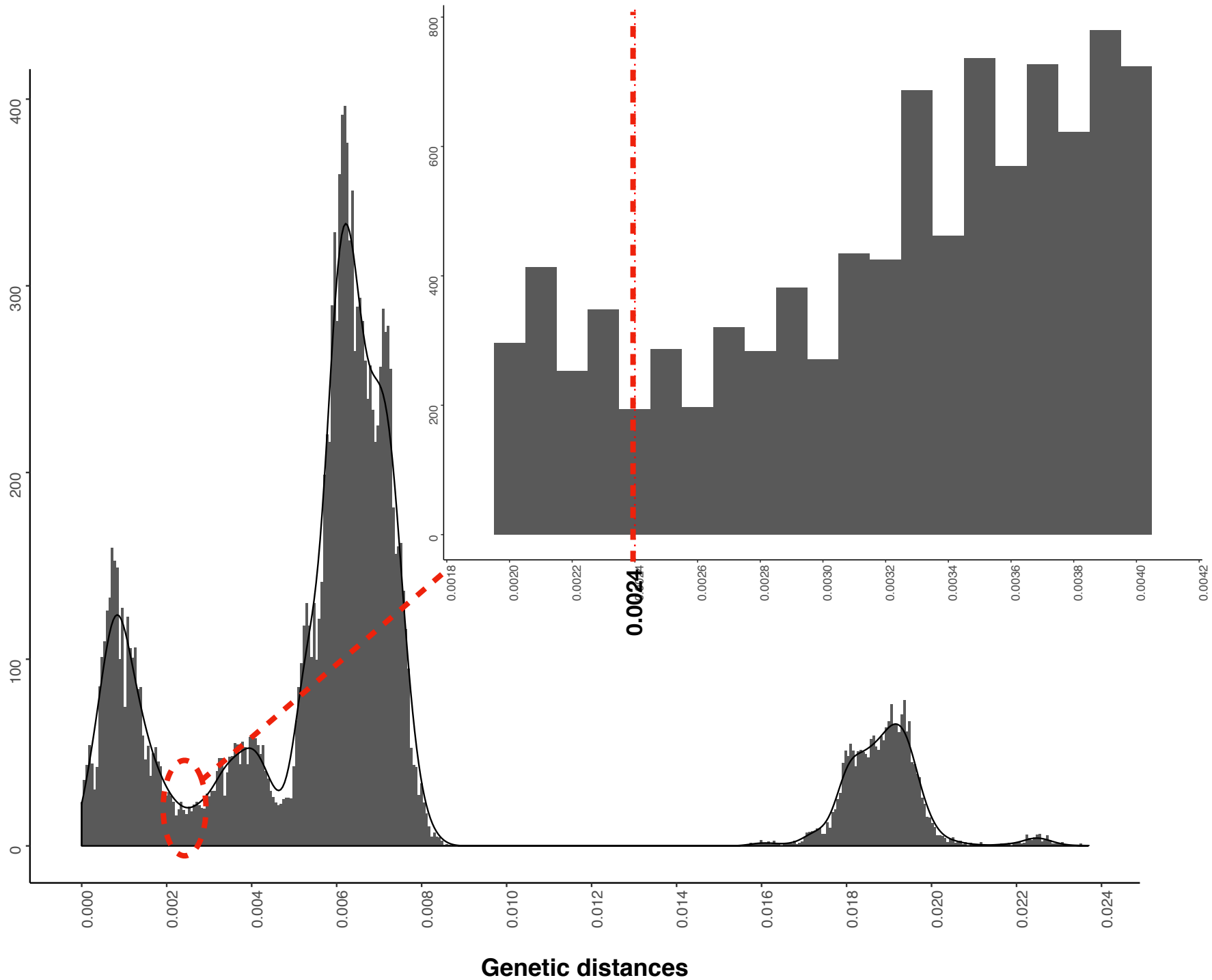

Supplement: veaa050_Supplementary_Data [file ve_6_2_veaa050_s6.zip › FigS1.pdf]
